# Halogenation of glycopeptide antibiotics occurs at the amino acid level during non-ribosomal peptide synthesis†
†Electronic supplementary information (ESI) available: Southern blot analyses of wildtype and the truncation mutants CK2.1 and CK2.2; LC-MS analysis of dipeptides from *in vivo* expression experiments together with authentic standards; SDS-PAGE and spectral analysis of Tcp21 and BhaA halogenase enzymes. See DOI: 10.1039/c7sc00460e



**DOI:** 10.1039/c7sc00460e

**Published:** 2017-07-13

**Authors:** Tiia Kittilä, Claudia Kittel, Julien Tailhades, Diane Butz, Melanie Schoppet, Anita Büttner, Rob J. A. Goode, Ralf B. Schittenhelm, Karl-Heinz van Pee, Roderich D. Süssmuth, Wolfgang Wohlleben, Max J. Cryle, Evi Stegmann

**Affiliations:** a Department of Biomolecular Mechanisms , Max Planck Institute for Medical Research , Jahnstrasse 29 , 69120 Heidelberg , Germany; b Interfaculty Institute of Microbiology and Infection Medicine Tuebingen , Microbiology/Biotechnology , University of Tuebingen , Auf der Morgenstelle 28 , 72076 Tuebingen , Germany . Email: evi.stegmann@biotech.uni-tuebingen.de; c EMBL Australia , Monash University , Clayton , Victoria 3800 , Australia . Email: max.cryle@monash.edu; d The Monash Biomedicine Discovery Institute , Department of Biochemistry and Molecular Biology , Monash University , Clayton , Victoria 3800 , Australia; e Institut für Chemie , Technische Universität Berlin , 10623 Berlin , Germany; f Allgemeine Biochemie , TU Dresden , 01062 Dresden , Germany; g Monash Biomedical Proteomics Facility , Monash University , Clayton , Victoria 3800 , Australia; h German Centre for Infection Research (DZIF) , Partner Site Tuebingen , Tuebingen , Germany; i ARC Centre of Excellence in Advanced Molecular Imaging , Monash University , Clayton , Victoria 3800 , Australia

## Abstract

Halogenation plays a significant role in the activity of the glycopeptide antibiotics (GPAs), although up until now the timing and therefore exact substrate involved was unclear. Here, we present results combined from *in vivo* and *in vitro* studies that reveal the substrates for the halogenase enzymes from GPA biosynthesis as amino acid residues bound to peptidyl carrier protein (PCP)-domains from the non-ribosomal peptide synthetase machinery: no activity was detected upon either free amino acids or PCP-bound peptides. Furthermore, we show that the selectivity of GPA halogenase enzymes depends upon both the structure of the bound amino acid and the PCP domain, rather than being driven solely *via* the PCP domain. These studies provide the first detailed understanding of how halogenation is performed during GPA biosynthesis and highlight the importance and versatility of *trans*-acting enzymes that operate during peptide assembly by non-ribosomal peptide synthetases.

## Abstract 2

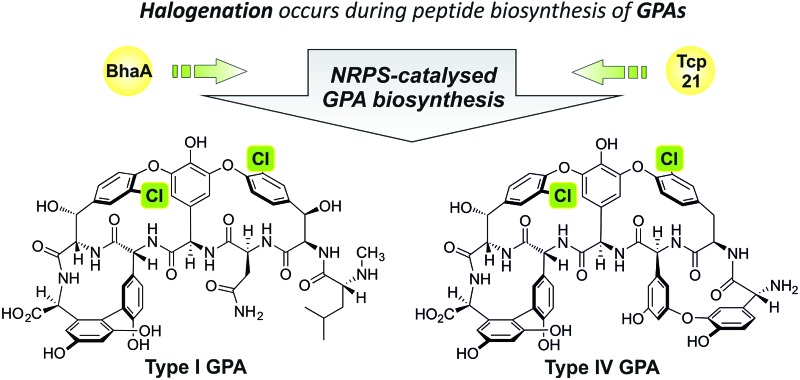
Halogenase enzymes involved in glycopeptide antibiotic biosynthesis accept aminoacyl-carrier protein substrates.

## Introduction

Non-ribosomal peptide synthesis is a highly important route for the production of bioactive peptides, many of which have medicinal value. Some of the most important members of non-ribosomal peptides are known for their antibiotic action: well-known examples include beta-lactam antibiotics such as nocardicin, cyclic peptides such as daptomycin and the glycopeptide antibiotics (GPAs) that include vancomycin and teicoplanin.^1^ By decoupling peptide synthesis from the ribosome, non-ribosomal peptide synthetase (NRPS) machineries are able to produce peptides from a significantly larger pool of precursors than the standard proteinogenic amino acids.^2,3^ NRPS-catalysed biosynthesis is based around active domains that are organised into modules. Adenylation (A)-domains catalyse the selection and activation of precursors, which are then loaded onto peptidyl carrier protein (PCP)-domains *via* a phosphopantetheine linker.^4^ The resultant thioester linkage remains chemically reactive for peptide bond formation – catalysed by condensation (C)-domains – but retains NRPS biosynthetic intermediates in a protein-bound form.^5^ In addition to these domains crucial for peptide synthesis, many NRPSs have additional modification domains, such as epimerisation (E)-domains, which alter the stereochemistry of amino acid residues and are a common hallmark of NRPS-produced peptides.^6^ The final peptide product is detached from the NRPS machinery *via* hydrolysis or cyclisation catalysed by a thioesterase (TE)-domain, which also offers further options to gain biological activity and compound diversity.^7–9^

The complexity of the products of NRPS biosynthesis not only stems from the direct actions of the NRPS assembly line but also from a great number of further peptide modifications by external enzymes acting in *trans*. Examples from GPA biosynthesis include the hydroxylation of amino acid side chains, cytochrome P450-catalysed crosslinking of aromatic side chains, glycosylation, sulfation, acylation as well as the incorporation of chlorine atoms into the peptide *via* the halogenation of aromatic side chains of amino acid residues.^10^ Many of the modifications appear to be directed towards improving the selectivity of GPAs towards specific bacterial strains but some, for example the installation of crosslinks into the peptide backbone, are essential for their antibiotic activity.^10,11^ Because of their crucial roles in GPA biosynthesis, the modification enzymes are an appealing target for GPA remodelling.^12^

**Fig. 1:**
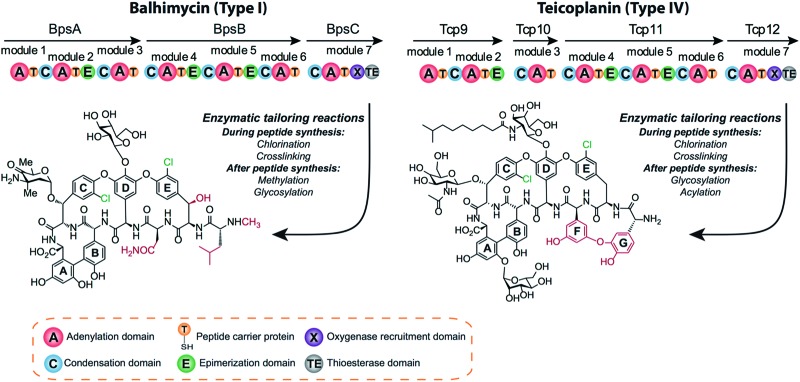
Biosynthesis of glycopeptide antibiotics. Biosynthesis of balhimycin (Type I GPA) and teicoplanin (Type IV GPA) is highly similar: their peptide backbones are produced by modular nonribosomal peptide synthetase machineries (major structural differences in the structures of the peptide backbones are indicated in red). The peptides produced are modified by crosslinking and chlorination (indicated in green) while the peptide is still bound to the machinery and by glycosylation after release of the peptide from the NRPS machinery. Teicoplanin is furthermore decorated by acylation and balhimycin by methylation. The numbering of each of the peptide residues is shown in blue lettering.

GPAs can be divided into several classes based on the heptapeptide backbone structure.^10,13^ Type I GPAs possess aliphatic residues at positions one and three of the peptide and display three crosslinks between aromatic residues of the peptide, forming the AB, C-*O*-D and D-*O*-E rings needed for GPA activity. Type II GPAs share the crosslinking pattern of Type I GPAs but contain aromatic residues at positions one and three of the peptide. Type III and IV GPAs also have aromatic residues at positions one and three of the peptide, but these are crosslinked to form an additional F-*O*-G ring, with differentiation between these classes due to the presence of an acyl moiety in Type IV GPAs that is absent in Type III GPAs. Balhimycin belongs to the vancomycin-type peptides (Type I GPAs), with balhimycin differing from vancomycin only with regard to the glycosylation pattern, whilst teicoplanin belongs to the Type IV GPAs (Fig. 1).

The chlorine atoms present within the GPA peptides contribute significantly to the binding affinity of the GPAs for their target, the dipeptide terminus of lipid II.^14–16^ In both Type I and IV GPAs β-hydroxy-tyrosine (OH–Tyr) and/or tyrosine (Tyr) residues are chlorinated in the positions two and six by halogenases that belong to the class of flavin (FADH_2_)-dependent halogenases. Most GPA gene clusters encode a single halogenase, which was shown in balhimycin biosynthesis to be responsible for chlorination of both positions in the peptide.^17^ Some GPA gene clusters possess an additional halogenase enzyme that appears to play a role in the chlorination of 4-hydroxyphenylglycine (4-Hpg) residues.^18^ Whilst insights into the mechanism of FADH_2_-dependent halogenases have been gained from structural and functional studies,^19,20^ reconstitution of halogenase activity *in vitro* has proven to be difficult. Most of the FADH_2_-dependent halogenases characterised so far are tryptophan halogenases that act on substrates free in solution.^21–23^ However, halogenases acting on PCP-bound substrates have also been characterised.^24,25^ In both cases chlorination of the substrate takes place before monomer incorporation to the natural product.

**Fig. 2:**
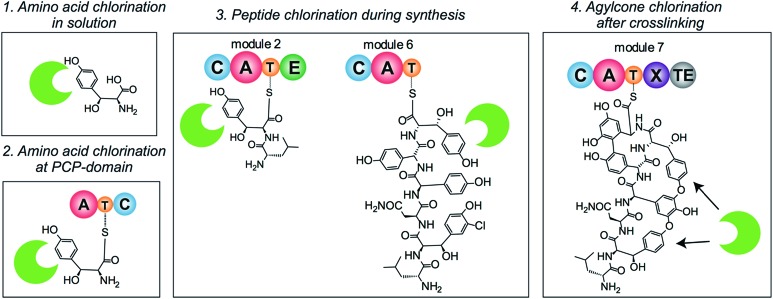
Possible scenarios for GPA halogenation. Four different scenarios for GPA chlorination have been suggested and to some extend studied. Halogenation of free amino acid has been ruled out but indications for the other three scenarios have been obtained.

The timing of the chlorination during GPA biosynthesis is still under debate. Four possible scenarios for the incorporation of halide atoms have been postulated and – to some extent – investigated (Fig. 2). In the first scenario, amino acids are chlorinated prior to incorporation by the NRPS machinery; in the second, halogenation of amino acids takes place after loading onto a PCP-domain; in the third, the peptide is chlorinated during peptide synthesis; and in the fourth, halogenation takes place after the peptide aglycone has been formed by P450 (Oxy) enzymes. Previously, the inactivation of the P450 monooxygenases responsible for GPA side chain cyclisation *in vivo* ^26–29^ strongly suggested that halogenation would take place prior to cyclisation. Furthermore, these scenarios have been studied using two homologous halogenases from Type I GPA biosynthesis: BhaA from balhimycin biosynthesis and VhaA from vancomycin biosynthesis. *In vivo* gene disruption studies of BhaA have shown that supplementation of the medium by chlorinated amino acids is not able to restore production of the natural, halogenated GPA if the halogenase gene is deleted.^30^ In contrast, supplementation of the growth medium with non-chlorinated OH–Tyr could overcome the deletion of the genes essential for OH–Tyr production and restore the formation of the halogenated type I GPAs.^30^ These results strongly suggest that scenario one, chlorination prior to incorporation by module 2 and 6 of the NRPS machinery, is not possible.

Although halogenation of free amino acids has been for most part ruled out, debate concerning the other three scenarios continues. The generation of various halogenated peptide GPA intermediates in a strain where the final condensation domain had been deleted points towards incorporation of the halide atoms during peptide biosynthesis.^11^ Precedent would suggest a mechanism of action similar to that identified for the halogenase enzymes from pyoluteorin synthesis^24^ or enediyne synthesis,^25^ where aminoacyl-PCP domains are the substrates for modification during peptide biosynthesis by the NRPS machinery; other enzymes have also been identified that adopt this approach.^31,32^ In contrast to this, *in vitro* studies using a P450 enzyme that crosslinks aromatic side chains from the vancomycin biosynthetic cluster (OxyB_van_) showed that halide atoms in PCP-bound hexapeptide substrates made the peptides worse substrates for the first crosslinking step,^33^ suggesting that chlorination would take place on the aglycone. However, these studies did not take into account the role of the X-domain in recruiting the P450 enzymes to the NRPS-bound peptide^34–38^ and recent results^39^ indicate that halogenated peptides are crosslinked equally well when presented by NRPS modules containing the X-domain, making halogenation prior to P450 activity a distinct possibility.

So far, all characterised FADH_2_-dependent halogenases associated with natural products produced by NRPS machineries act on amino acid/pyrrole substrates rather than on the final product, strongly suggesting the PCP-bound amino acid to be the substrate for halogenases in GPA biosynthesis. However, *in vitro* experiments using VhaA showed low levels of activity upon PCP-bound hexapeptide substrates^40^ pointing towards chlorination of the peptide rather than amino acid substrate. Thus, the exact timing of halogenation in GPA biosynthesis has remained unclear until this current study. Here, we are now able to show that halogenation occurs during peptide biosynthesis and that the substrates for the GPA halogenase enzymes are the PCP-bound amino acids, two OH–Tyr residues at amino acid (AA) position 2 and 6 for Type I GPA and two Tyr residues at position 2 and 6 for Type IV GPAs.

## Results

### Analysis of GPA halogenation *in vitro*

In order to assess the halogenation state of peptide precursors during GPA biosynthesis, we performed *in vitro* turnover experiments. In this regard, we explored the halogenase enzymes from both the balhimycin producer (*Amycolatopsis balhimycina*) as well as the teicoplanin producer (*A. teichomyceticus*). Both enzymes could be overproduced and purified in an FAD-bound form as anticipated (Fig. S1†). The former enzyme was overproduced in *Pseudomonas fluorescens* BL915 ΔORF1-4 containing the gene *bhaA* ^40^ in the *E. coli-Pseudomonas* shuttle vector pCIBhis and the latter in *E. coli*.

We prepared a range of substrates for activity assays: tyrosine, Type IV dipeptide (4-Hpg-Tyr), hexapeptide^41^ (leucine (Leu)-Tyr-asparagine (Asn)-4-Hpg-4-Hpg-Tyr) and two different heptapeptides^42^ (Leu-Tyr-Asn-4-Hpg-4-Hpg-Tyr-Dpg and 4-Hpg-Tyr-Dpg-4-Hpg-4-Hpg-Tyr-Dpg) (Fig. 4–6). Substrates were loaded on various PCP domains using the promiscuous phosphopantetheinyl transferase Sfp. Tyrosine was loaded on both PCP-domains that would be expected to interact with the halogenase enzyme (PCP2 and PCP6 from a Type IV NRPS) as well as to a PCP-domain that – based on the GPA chlorination pattern – is not anticipated to interact with the halogenase enzyme (PCP1 from a Type IV NRPS) to test if the PCP-domain is responsible for guiding the halogenase to the correct substrate. Dipeptide was loaded onto a PCP2 domain (Type IV) and hexapeptide on a PCP7 domain (Type I), whilst heptapeptides were loaded on PCP7 domain either from Type I or Type IV NRPSs. Chlorination of tyrosine and dipeptide were also studied using substrates free in solution. We assessed activity using UPLC-MS with single ion monitoring (SIM) and multiple reaction monitoring (MRM), after cleavage of PCP-bound substrates using excess methylamine to afford the methylamide products.

**Fig. 3:**
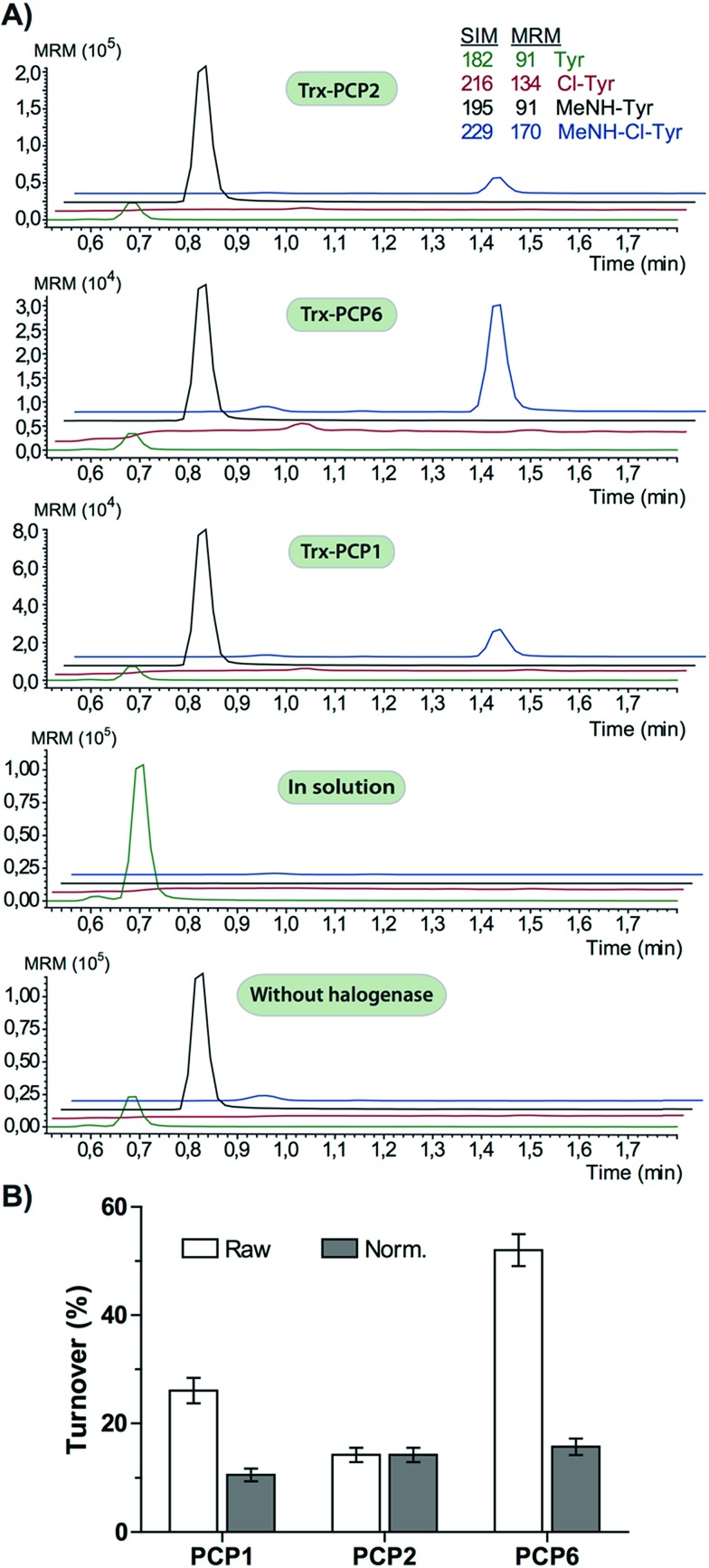
Chlorination of tyrosine substrates by Tcp21. (A) Chlorination of tyrosine bound to PCP-domains 1, 2 and 6 (Type IV NRPS) was detected but not for tyrosine in solution. (B) Turnover levels appear to vary between different PCP domains (raw) but after normalizing turnover level with loading efficiency it is apparent that the turnover level is not affected by the identity of the PCP domain (norm). Loading of PCP2 was set to 100%. SIM = single ion monitoring, MRM = multiple reaction monitoring. Colours indicate Tyr (green/black) and chloro-Tyr (red/blue) as acids/methylamides.

**Fig. 4:**
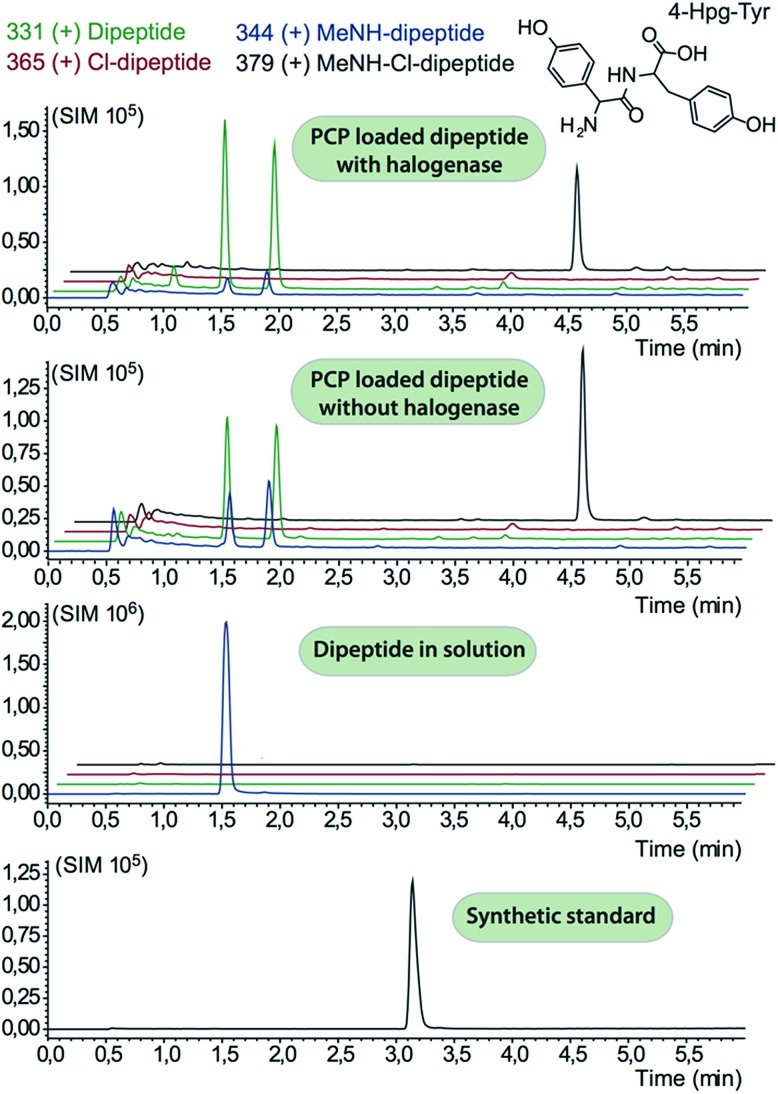
Analysis of dipeptide chlorination by Tcp21. Traces show two peaks for the non-chlorinated peptide due to racemisation during peptide synthesis. A peak with the same *m*/*z* ratio as methylamide-Cl-dipeptide is seen in both turnover and control reactions: however, the retention time of this peak does not correspond with authentic chlorinated dipeptide standards indicating that this peak does not correspond to chlorinate dipeptide and is unrelated to enzymatic activity; no peaks for chlorinated products were detected.

**Fig. 5:**
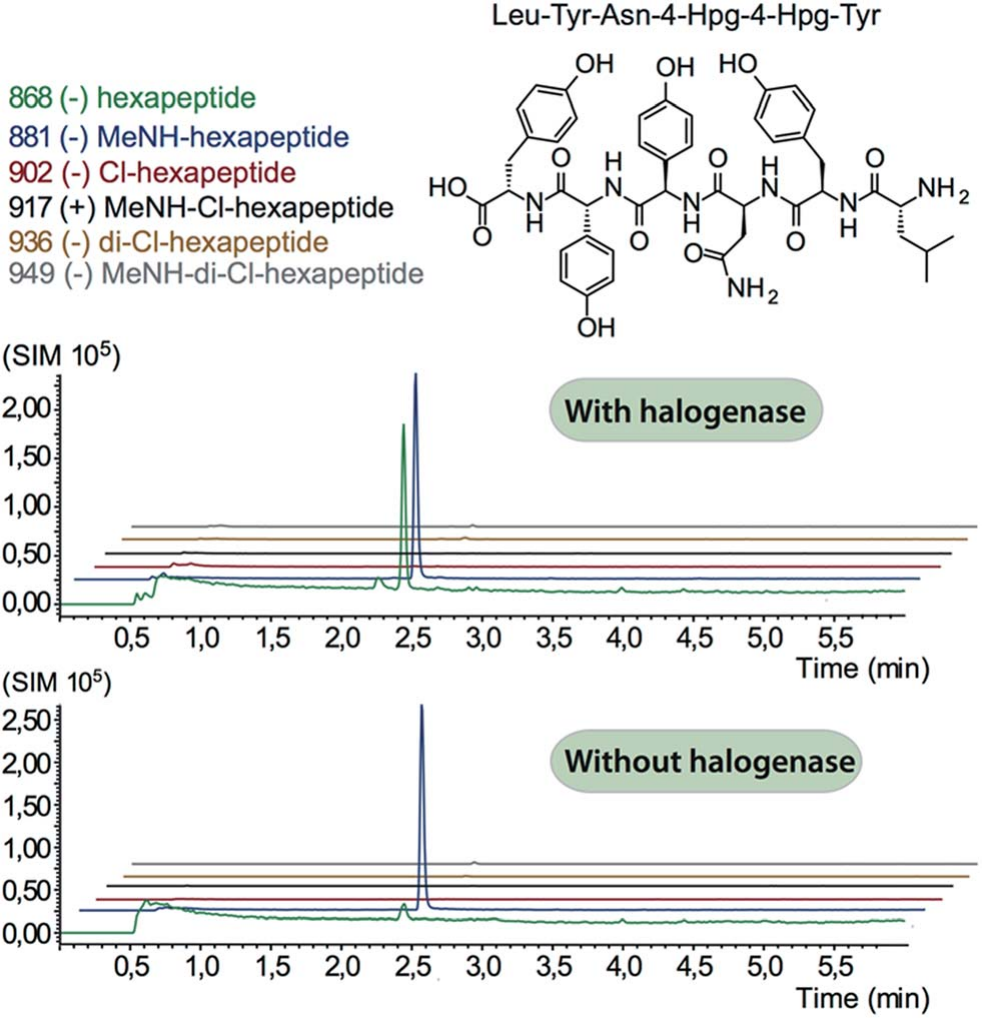
Analysis of hexapeptide chlorination by Tcp21. No enzymatic activity was detected, with only hydrolysed and methylamine cleaved non-chlorinated peptides detected. The structure of the peptide used is shown in the top panel.

**Fig. 6:**
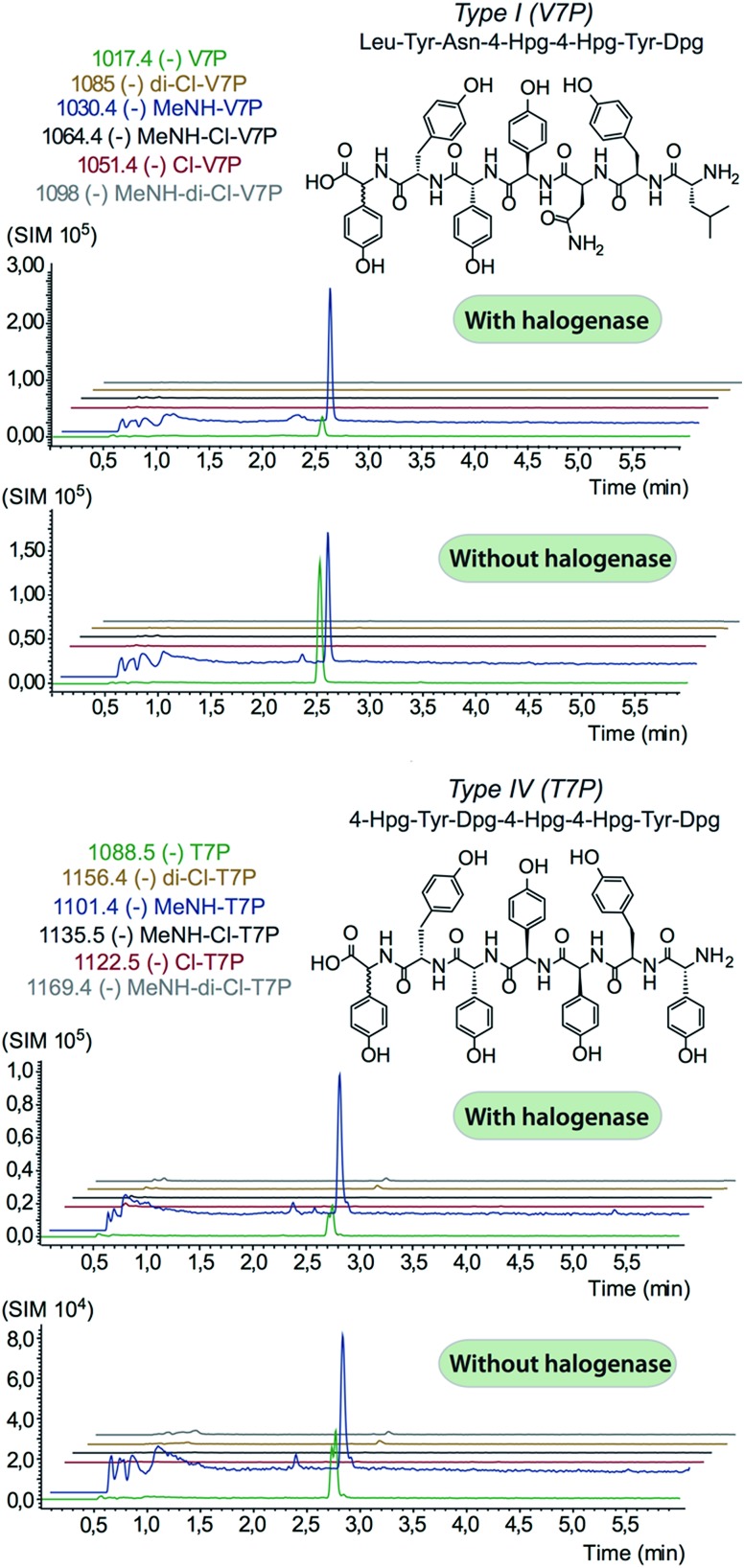
Analysis of heptapeptide chlorination by Tcp21. No enzymatic activity was detected for either type-I (upper panel) or type-IV (lower panel) peptide structures, with only hydrolysed and methylamine cleaved non-chlorinated peptides detected. Structures of the peptides are indicated in the relevant panels of the figure.

The halogenase from the teicoplanin system (Tcp21) displayed activity upon PCP-bound tyrosine as a substrate. We could observe activity upon tyrosine bound to PCP-domains 1, 2 and 6 from the teicoplanin NRPS machinery, which implies that the halogenase is not solely selective for the PCP domain and that the bound amino acid plays a role in regulating halogenase activity (Fig. 3A). Tyrosine in solution was not chlorinated by Tcp21, which suggests that the interaction with a PCP domain is crucial for catalysis. The loading efficiency differed between the three PCP-domains but the amount of chlorinated tyrosine stayed at the same level in each reaction, which shows that the turnover efficiency appears to be limited by the *in vitro* efficiency of Tcp21 (Fig. 3B). We also tested whether the halogenase was able to act upon dipeptide substrates. Under the conditions where we observed halogenase activity upon PCP-bound tyrosine, we could show that neither PCP-loaded nor free dipeptide was a substrate for the enzyme (Fig. 4). We also tested longer peptides as potential substrates for halogenation, however – and as with the dipeptide substrates – we did not observe any activity upon these substrates (Fig. 5 and 6). Thus, the results of our *in vitro* characterisation indicate that PCP-bound amino acids are the actual substrates for halogenation during Type IV GPA biosynthesis. We did not detect activity of the balhimycin halogenase (BhaA) upon any substrates (data not shown), which we attribute to the lack of the hydroxylated side chain of the tyrosine residue (present in Type I GPAs) in the aminoacyl-PCP substrate.^10^

### Construction of a dipeptide producing GPA strain

**Fig. 7:**
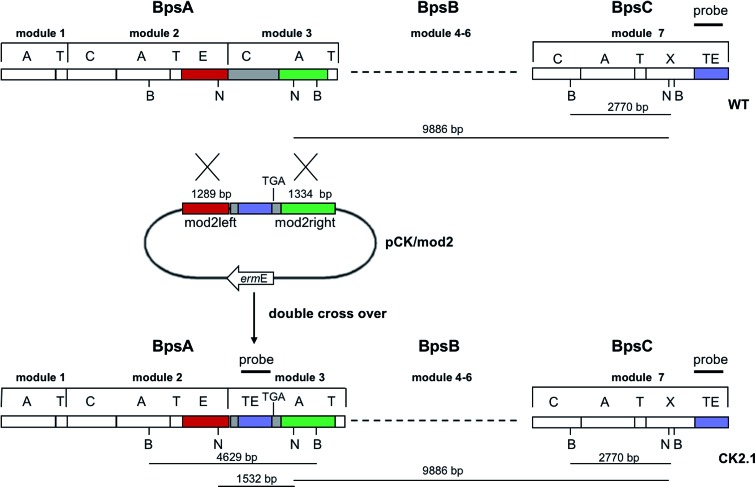
Construction of the mutant CK2.1. Schematic representation of the insertion of a TE-encoding DNA fragment into *bpsA*. Abbreviations for the domains see Fig. 1. WT, *A. balhimycina* wildtype; *ermE*, erythromycin resistance gene. Mod2left (1289 bp), containing the E-domain (red) and an N-terminal part of the C-domain (grey), and mod2right (1334 bp) including the A-domain (green) and a C-terminal part of the C-domain (grey), TE fragment (blue). To obtain the replacement mutant CK2.1 a double crossover *via* homologous recombination is required (see Results). Line: fragments after NcoI (N) or BamHI (B) restriction.

Since BhaA showed no *in vitro* activity, we decided to assess the halogenation state in *A. balhimycina in vivo*. Given that we have elucidated balhimycin biosynthesis in detail,^12^ have developed tools to construct *A. balhimycina* mutants and elucidated the resultant intermediates, we generated a balhimycin producer strain that possessed a modified NRPS machinery to investigate halogenation.^43–45^ As our hypothesis is that the PCP-bound amino acids are substrates for the halogenase during GPA biosynthesis, we modified the balhimycin NRPS in such a way as to address the first peptide intermediate that should contain a halogenated residue – a dipeptide. Three NRPS proteins, BpsA, BpsB and BpsC, are involved in the assembly of the GPA heptapeptide core (Fig. 7). Module 2 is part of BpsA and consists of C-A-PCP (PCP1) and E domains, while module 3 consists of a C-, A- and PCP-domain (PCP3). The C-domain of module 3 is responsible for tripeptide formation from the Leu–OH–Tyr dipeptide and Asn. To terminate the non-ribosomal peptide synthesis after module 2 and hence produce a dipeptide for analysis, significant parts of the C-domain of module 3 were deleted and replaced by TE-domain of BpsC to provoke release of the dipeptide intermediate (Fig. 7). To this end, the fragments mod2left (1289 bp), containing the E-domain and an N-terminal part of the C-domain of module 3, and mod2right (1334 bp) including the C-terminal part of the C-domain and the A-domain of module 3, were amplified by PCR and used for the deletion of the C-domain (see Experimental procedures, Fig. 7).

The TE-domain of BpsC was amplified by PCR and inserted between the fragments mod2left and mod2right resulting in the plasmid pCKmod2. The non-replicative plasmid pCK/mod2 was used to transform the *A. balhimycina* wildtype strain by the direct transformation method. The plasmid selection marker conferring erythromycin resistance was used to identify clones in which a first homologous recombination event occurred. Ten erythromycin resistant clones were obtained. These clones were analysed in a bioassay with *Bacillus subtilis* ATCC6633, to check whether the integration of pCK/mod2 resulted in the loss of balhimycin production. Loss of balhimycin production would be the expected result of recombination *via* the chromosomal *bpsA* gene. Two of the ten clones lost the ability to produce an active antibiotic and the correct integration of pCKmod2 was verified by Southern Blot hybridisation; both clones turned out to be identical. The non-producing clone (CK1) showed the intended integration of the plasmid within the region coding for the C-domain. For the other eight erythromycin resistant clones that still produced balhimycin the crossover took place *via* the part of the *bpsC* gene which encodes the TE-domain at the end of BpsC.

To obtain the replacement mutant, a second homologous recombination in CK1 was required: since double crossover events happen at a low frequency in *A. balhimycina*, a “stress” treatment (see Experimental procedures) – whose application increases the probability of a second crossover event – was carried out with clone CK1. Following this protocol, 650 colonies were examined on R5 agar plates both in the presence and absence of erythromycin. Two of the “stressed” colonies lost the integrated plasmid as indicated by the lack of erythromycin resistance. These two clones, CK2.1 and CK2.2, were not able to produce an active antibiotic, indicating that the second crossover led to the disruption of the non-ribosomal peptide biosynthesis. The gene replacement and loss of pCK/mod2 was confirmed by Southern Blot analyses; a DIG-labelled PCR fragment of the TE-domain was used as a probe with total DNA of the two mutants CK2.1 and CK2.2 and the wildtype of *A. balhimycina*. The Southern Blot clearly showed two bands of the expected size in the case of the mutants while in the total DNA of *A. balhimycina* wildtype only a single band was visible (Fig. S2†). This result combined with the loss of balhimycin production showed that CK2.1 and CK2.2 are identical and confirmed the correct identity of the replacement mutants.

The mutant CK2.1 was fermented under production conditions and the culture broth was analysed by HPLC-ESI-MS. In accordance to the lack of antibiotic activity in the bioassays, no balhimycin was detected. In contrast, the search for biosynthetic intermediates resulted in signals that corresponded to Leu–OH–Cl-Tyr (Fig. S3†). Remarkably, the molecular mass of this dipeptide was found at two different retention times in the HPLC run, which suggests the presence of two diastereoisomers due to alternate stereochemistry of the second amino acid, β-hydroxytyrosine (Fig. S3†). Given that the epimerisation domain that converts (l)–OH–Tyr to (d)–OH–Tyr in the NRPS is still present, this implies that the inserted thioesterase domain is able to compete effectively with the E-domain for the peptidyl-PCP substrate to generate two diastereomers of the product dipeptide. In order to confirm the constitutional structures of these dipeptides, daughter ion spectra were generated and signals assigned. It was found that the main fragment signals of both diastereoisomers were identical indicating the same constitutional formulae (Fig. S4†). We further synthesised synthetic standards of the suspected Leu–OH–Cl-Tyr diastereomers and could thus confirm the identity of the products identified from the *A. balhimycina* mutant, with the major dipeptide peak corresponding to the dipeptide bearing the (l)–OH–Tyr residue (Fig. S5 and S6†). This result confirms that halogenation during GPA biosynthesis already takes place on module 2 during NRPS synthesis.

## Discussion

The diversification of peptide scaffolds within NRPS biosynthesis provides a tremendous source of selectivity and function. Halogenation is an important example of one such modification, and the GPAs are an excellent example of the effects halogenation can have on compound activity, as the removal of chlorine atoms from GPAs leads to significant reduction in binding affinity for the dipeptide terminus of lipid II.^14–16^ Halogenase activity from different biosynthetic systems has been demonstrated for different types of substrates – either free in solution or carrier protein bound – with selectivity varying from one system to another.^46^ However, the timing and substrate selectivity of halogenation during GPA biosynthesis has previously been unclear. Whilst the activity of the vancomycin halogenase upon PCP-bound peptides has been reported, the use of mismatched peptide and PCP-domain within the substrates tested and the lack of cohesion with previous preliminary *in vivo* data led us to investigate this process further.^40^ In this work, we have been able to combine comprehensive results of both *in vivo* and *in vitro* experiments to show that GPA halogenase enzymes act during NRPS-mediated peptide assembly. Initially, through the *in vitro* characterisation of the halogenase Tcp21 from teicoplanin biosynthesis we could clearly demonstrate that halogenase activity was present with tyrosine only when the amino acid was presented in a PCP-bound form. Furthermore, we could show that the identity of the PCP-domain able to support tyrosine halogenation was not strictly correlated with the observed halogenation pattern of teicoplanin, which suggests that a combination of correct amino acid and PCP-domain is required for GPA halogenation during NRPS-catalysed peptide synthesis *in vivo*. Our results are in contrast to the results described by Schmartz *et al.*,^40^ in which the authors demonstrated the chlorination of both OH–Tyr residues on a linear hexapeptide intermediate attached to the PCP domain in module-6 of the NRPS during vancomycin assembly. Chlorination at this stage of GPA biosynthesis shows that this halogenase demonstrates the *in vitro* capability to modify two quite different sites within the hexapeptide-PCP substrate: this activity is challenging to reconcile with other examples of GPA halogenation (*e.g.* A40926,^47^ which displays chlorination at residues 3 and 6 of the peptide, or the significantly altered pattern found in the Type-V GPA complestatin).^48^ We therefore decided to explore our hypothesis that chlorination occurs on the PCP-bound amino acid using further *in vitro* and *in vivo* studies on a different GPA halogenase: the balhimycin halogenase BhaA. Previous *in vivo* gene disruption experiments of the balhimycin synthetase had revealed the presence of chlorinated GPA peptide precursors during NRPS-catalysed peptide synthesis.^11^ In order to further test whether the substrates of GPA halogenation are aminoacyl-PCPs, we constructed a mutant balhimycin producer strain that had been engineered to produce the minimal GPA peptide that could contain a chlorine atom – a dipeptide. Following the successful construction of the strain, analysis of the peptide products and comparison to authentic standards clearly revealed the presence and confirmed the identity of the chlorinated dipeptides produced. This result indicates that halogenation during GPA biosynthesis occurs during NRPS-mediated peptide biosynthesis: as neither Tcp21 nor BhaA were active upon peptide substrates, the halogenase activity observed during NRPS-catalysed peptide synthesis *in vivo* can be deduced as occurring upon PCP-bound amino acids, which matches the results of our *in vitro* experiments. The lack of activity of BhaA upon PCP-bound tyrosine is also consistent with the differences in the generation of OH–Tyr residues during balhimycin and teicoplanin biosynthesis. In balhimycin biosynthesis, the correct PCP-bound amino acid substrates for BhaA would be OH–Tyr and not Tyr, as OH–Tyr is incorporated directly into the precursor peptide by the balhimycin NRPS machinery.^30,45^ This is significantly different to teicoplanin biosynthesis, in which tyrosine is initially recognised and incorporated into the GPA peptide by the NRPS and that is followed by subsequent generation of OH–Tyr *via* the actions of a non-heme iron oxygenase.^49^ The results from BhaA *in vitro* experiments further demonstrate the substrate specificity of the GPA halogenase enzymes and underlines the importance of having the correct amino acid loaded onto the PCP to enable halogenation during GPA biosynthesis.

## Conclusion

In this work, we have obtained conclusive evidence that halogenation occurs during NRPS-mediated peptide assembly in GPA biosynthesis. By combining the results of *in vitro* and *in vivo* experiments we have been able to show that aminoacyl-PCP substrates are recognised by halogenase enzymes and that the pairing of *both* the PCP-domain and bound substrate is crucial to activity: this model (see also ref. 50) helps to explain why some GPAs (such as A40926) display halogenation at different residues^47^ or differing levels,^51^ whilst others (such as pekiskomycin)^52^ are lacking chlorination at residues that would anticipate to be halogenated based on similar GPAs.^3^ Our GPA halogenation model also aligns with results obtained from biosynthetic studies of other halogenated peptides produced by NRPS-systems (such as enduracidin/ramoplanin), where switching the halogenase enzyme *in vivo* leads to a product where the halogenation pattern is altered in spite of highly similar peptide structures.^53,54^ Our results suggest that the alteration of halogenase enzymes within GPA producer strains could well produce alternatively halogenated GPA products: given that halogen atoms can act as handles for chemical modification of GPAs^55,56^ and the synthetic challenge of producing such compounds,^14,15^ switching halogenase enzymes (such as has been done for other modification enzymes in GPA biosynthesis)^57^ would appear to be an important route that should be explored to enable the future diversification of GPA scaffolds.

## Experimental

### *In vivo* experiments

#### Strains and plasmids

*E. coli* XL1-blue^58^ was used as general cloning host. *A. balhimycina* DSM5908 (ref. 59) is the balhimycin producing wildtype and was used to generate the NRPS mutant CK1 and CK2 (this study). The replacement plasmid pCKmod2 (this study) is a derivative of the non-replicative vector pSP1.^43^

#### Media and culture conditions

*Escherichia coli* strains were grown in Luria broth (LB) medium^60^ at 37 °C, supplemented with 100 μg ml^–1^ ampicillin when necessary to maintain plasmids. *A. balhimycina* strains were grown in R5 medium^61^ at 30 °C. Liquid/solid media were supplemented with 50 μg ml^–1^ erythromycin to select for strains carrying integrated antibiotic resistance genes. *P. fluorescens* was grown in LB medium at 30 °C in the presence of 50 μg ml^–1^ kanamycin and 30 μg ml^–1^ tetracycline for 72 h.

#### Preparation and manipulation of DNA

Methods for isolation and manipulation of DNA were performed as reported.^60,61^ PCR fragments were isolated from agarose gels with QIAquick gel extraction kit (Qiagen, Hilden, Germany). Restriction endonucleases (NEB, Ipswich, MA, USA and Fermentas, St. Leon-Rot, Germany) were used according to their specifications.

PCR protocols for amplification of the fragments mod2left, mod2right and TE PCRs were performed on a Robo Cycler Gradient 40 thermocycler from Stratagene (La Jolla, CA, USA) with the Expand High Fidelity PCR System (Roche, Grenzach-Wyhlen, Germany). For the amplification of the fragments mod2left, mod2right and TE the following PCR conditions were used: initial denaturation (95 °C for 5 min), 30 cycles of denaturation (95 °C for 2 min), annealing (69 °C for 1 min), and polymerisation (72 °C for 1 min 30 s), an additional polymerisation step (72 °C for 10 min) at the end. The primers used were as follows: amplification of mod2left (1289 bp): Mod2leftP1, Mod2leftP2; for amplification of fragment mod2right (1334 bp): Mod2rightP1, Mod2rightP2, and for TE (831 bp): TE2left, TE2right.

#### Construction of the replacement plasmid pCKmod2

Plasmid pCKmod2 was constructed for the interruption of the nonribosomal peptide synthesis after module 2 of BpsA. In addition to the deletion of the C-domain of module 3, the integration of a TE domain, (containing a stop codon to terminate translation of the downstream genes) should mediate the release of the synthesized intermediate. To this end, the fragments mod2left, mod2right and TE were amplified by PCR. Firstly, the TE fragment was cloned using blunt ends into the pDrive vector (Qiagen) (TE/pDrive). The second fragment mod2left was cloned into TE/pDrive using the restriction sites NdeI and XbaI (pDriveM2L/TE). Finally, the third fragment mod2right was cloned into pDriveM2L/TE following digestion with HpaI/SphI. The complete interruption cassette was cloned into the non-replicative vector pSP1 using XbaI and SphI to obtain pCKmod2 (scheme in Fig. 2).

#### Direct transformation of *A. balhimycina*

For transformation of *A. balhimycina*, a modified transformation method was used as described previously.^43^

#### “Stress” protocol

The stress treatment was essentially used as described previously.^17^ For further fragmentation, protoplast were generated as described by Thompson *et al.* ^62^ After storage on ice (10 min), 100 μl of appropriate dilutions (10^–1^ to 10^–4^) were plated on R5 agar plates. After incubation at 30 °C for 10–14 days, the colonies were used for further investigation.

#### Determination of balhimycin biosynthesis

Balhimycin production was determined by bioassays using *Bacillus subtilis* ATCC6633 as a test organism and cell-free supernatants of *A. balhimycina* strains grown in R5 medium.

#### Southern blot analyses

Digests of genomic *A. balhimycina* DNA were separated in 1% agarose gels in Tris-acetate buffer and transferred to Hybond-N Nylon membranes (GE Healthcare, München, Germany). For labelling of DNA probes and hybridisation the nonradioactive DIG DNA Labelling and Detection Kit from Roche was used at high stringency (0.1% SDS/0.1 × SSC, 68 °C). As a size standard, the DIG-labelled DNA Molecular Weight Marker VII (Roche) with the following fragment lengths (in base pairs) was used: 81, 359, 492, 710, 992, 1164, 1482, 1515, 1882, 1953, 2799, 3639, 4899, 6106, 7427 and 8576. The procedure followed a standard protocol.^60^

#### HPLC-ESI-MS and – MS-MS (culture broth)

The culture broth of the mutant CK1 was investigated by HPLC-ESI-MS(/MS). For sample preparation an adsorption chromatography with AMBERLITE® XAD16 material was performed. The used HPLC-ESI-MS-MS system consisted of a capillary-LC-system (1100 series, Agilent Technologies Deutschland GmbH, Böblingen, Germany) coupled to a QTrap2000 with a TurboIonSpray source (Applied Biosystems, Darmstadt, Germany). Separations were performed on a Jupiter 4 μm Proteo 90A column system (main column: 150 × 1 mm; precolumn: 30 × 1 mm; Phenomenex, Aschaffenburg, Germany) with a flow rate of 50 μl min^–1^ in micro mode and the following gradient: *t* = 0 min: 5% B; *t* = 10 min: 20% B; *t* = 13 min: 50% B; *t* = 14 min: 100% B (solvent A: 0.1% HCOOH in water, solvent B: 0.1% HCOOH in MeCN). The injection volume was 5 μl. The TurboIonSpray source dependent parameters were optimised for the used flow rate of 50 μl min^–1^ to: CUR 30, IS 5500, nebuliser gas 70, turbo gas 70, TEM 300. The compound dependent parameters were optimised with different glycopeptides to: DP 30, EP 12, CE 10, Q3 entry barrier 12. The EMS scans were carried out in positive mode, with a LIT scan rate of 1000 amu s^–1^ and dynamic fill time. The EPI scans had the following parameters: Q1 resolution unit, LIT scan rate 1000 amu s^–1^, fixed LIT fill time 500 ms, CE 30, CES 20, CAD gas high.

#### HRMS and MS2 analyses for synthetic dipeptides

Samples were diluted in 40% MeCN/0.1% formic acid and infused at 3 μl min^–1^ through a H-ESI source into an Orbitrap Fusion Tribrid mass spectrometer (Thermo Scientific). Instrument settings were source voltage 3500 V, sheath gas 2.0, ion transfer tube temp 300, collision pressure 0.008. Full MS scans were acquired with resolution 500k; scan range: 150–2000; AGC target 1 × 10^6^. High resolution MS^2^ spectra were acquired for the 345.12 *m*/*z* singly charged ion with the following settings: Orbitrap resolution: 60k; isolation window: 2.0 *m*/*z*; HCD Collision Energy: 10. Acquired.raw files were analyzed in XCalibur Qual Browser (Thermo Scientific).

### *In vitro* experiments

#### Cloning

Synthetic genes encoding *tcp9*, *tcp11* and *tcp21* (*selected sequence range: 16-506*) from *A. teichomyceticus* (DSMZ 43 866, Eurofins Genomics MWG) and *ppat* (phosphopantetheine adenylyl transferase) from *E. coli* (Geneart) were codon-optimised for expression in *E. coli*. The *panK* gene (encoding pantothenate kinase) was amplified directly from *E. coli* strain DH10β using whole cell PCR, the primers in Table 1 and GoTaq® Green Premix (Promega). The *tcp21* and *ppat* genes were cut out from the pEX-K plasmid using NdeI and HindIII restriction enzymes (New England Biolabs). The areas encoding PCP1 and PCP2 from the *tcp9* gene and PCP6 from the *tcp11* gene were amplified by PCR using Phusion® high fidelity polymerase (NEB) and the primers indicated in Table 1. The genes for PCP domains were subcloned into a modified pET vector^63^ containing under a T7 promoter the thioredoxin solubility tag followed by a TEV cleavage site and an N-terminal His6-tag. The PCR products and the vector were cut using NcoI and XhoI restriction digest (FastDigest, ThermoFisher Scientific), ligated with T4 ligase (ThermoFisher Scientific) and transformed to BL21-Gold (DE3) cells. The genes encoding Tcp21, PanK and PPAT were subcloned into pET-28(a) vector (Novagen). The inserts and vector were cut using NdeI and HindIII-HF restriction enzymes, ligated with T4 ligase and transformed into Bl21-Gold (DE3) cells. The *B. subtilis* YcnD (NADH-dependent oxidoreductase) expression plasmid was a kind gift from Prof. P. Macheroux.^64^ Protein constructs for GB1-PCP7_cep_ and GB1-PCP_tei_ are described in Haslinger and Peschke *et al.* ^36^

**Table 1:** Primer names and sequences. Restriction sites underlined

Primer name	Sequence
PCP1_fwd	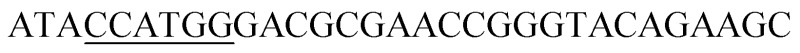
PCP1_rev	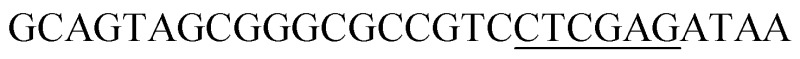
PCP2_fwd	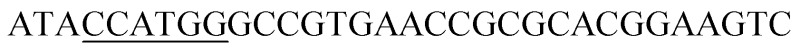
PCP2_rev	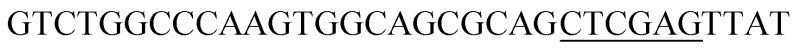
PCP6_fwd	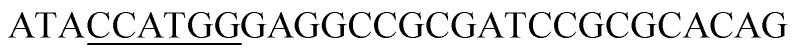
PCP6_rev	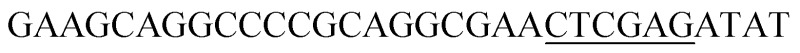
PanK_fwd	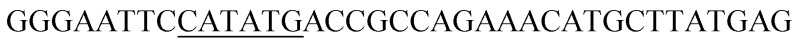
PanK_rev	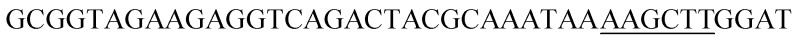
Mod2rightP1	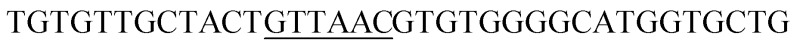
Mod2rightP2	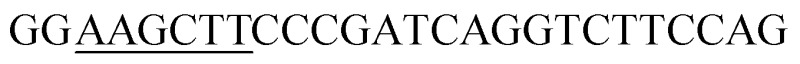
Mod2leftP1	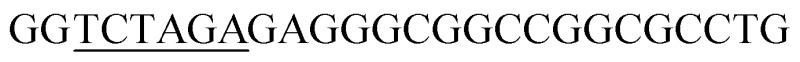
Mod2leftP2	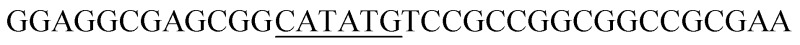
TE2left	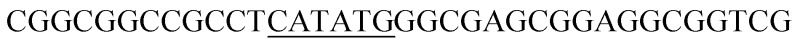
TE2right	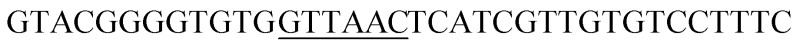

#### Gene expression and protein purification

LB-medium was supplemented with kanamycin (50 mg ml^–1^) and inoculated with an overnight culture (1% *E. coli*). Cells were grown to OD_600_ = 0.5–0.8 (37 °C, 80 rpm) and gene expression was induced with 0.1 mM IPTG. Cells were grown over night (18 °C, 80 rpm) and harvested by centrifugation (5500*g*, 10 min, 4 °C). Cell pellets were resuspended in lysis buffer (50 mM Tris·HCl, pH 7.4, 50 mM NaCl, 10 mM imidazole), flash frozen and stored at –80 °C. All purification steps were performed at 4 °C or on ice except for purification steps on an Äkta system that were performed at RT. Before lysis, a protein inhibitor cocktail tablet (EDTA free, Sigma-Aldrich) was added to the thawed cells. Cells were lysed *via* four passes through a Microfluidizer (Microfluidics) and the lysate was clarified by centrifugation (38 800*g*, 1 h). Supernatant was incubated with Ni-NTA beads (1 h, Macherey-Nagel) with gentle shaking and the beads were washed twice with NiNTA wash buffer (50 mM Tris·HCl, pH 7.4, 300 mM NaCl, 10 mM imidazole). Protein was eluted with NiNTA elution buffer (50 mM Tris·HCl, pH 7.4, 300 mM NaCl, 300 mM imidazole). Size exclusion chromatography (SEC) was performed using a Superose 12 10/300 GL column connected to an Äkta PURE system (GE Healthcare). Protein concentration was determined by spectroscopic method (A_280_, Nanodrop 2000c, Nanodrop) and protein concentrated using centrifugal concentrators (Sartorius). Protein purity was assessed by SDS-PAGE and concentrated proteins were aliquoted (30–100 μl), flash frozen and stored at –80 °C. Identity of the purified proteins was confirmed by MALDI-TOF MS peptide mass fingerprinting.

#### Details for specific purifications

Halogenase: 2 ml NiNTA beads were incubated with the cleared lysate. NiNTA elution fraction was dialysed against 50 mM Tris·HCl, pH 7.1, 50 mM NaCl and concentrated to 20 mg ml^–1^ (calculated *ε* = 88 790 M^–1^ cm^–1^ was used to determine protein concentration). YcnD: 4 ml NiNTA beads were incubated with the cleared lysate. Elution fraction was dialysed against 50 mM Tris·HCl, pH 7.4, 20 mM NaCl and subjected to anion exchange purification (Resource Q, GE Healthcare). Appropriate fractions were pooled and subjected to SEC (50 mM Tris·HCl, pH 7.4, 150 mM NaCl). Fractions containing monomeric YcnD were pooled and concentrated to 17 mg ml^–1^ (calculated *ε* = 36 910 M^–1^ cm^–1^ was used to determine protein concentration). Trx-PCP1, Trx-PCP2 and Trx-PCP6: 2 mM dithioerythritol (DTE) was added to cells and to all purification buffers. 5 ml NiNTA beads were added to the cleared lysate. NiNTA elution fraction was further purified by SEC (50 mM Tris·HCl, pH 7.4, 150 mM NaCl, 1.5 mM TCEP). Appropriate fractions were pooled and concentrated to approximately 15 mg ml^–1^ (calculated *ε*: Trx-PCP1: 22 234.4 M^–1^ cm^–1^, Trx-PCP2: 22 607 M^–1^ cm^–1^ and Trx-PCP6: 20 970 M^–1^ cm^–1^ were used to determine protein concentration). PanK: 4 ml NiNTA beads were added to the cleared lysate. NiNTA elution fraction was further purified by SEC (50 mM Tris·HCl, pH 7.4, 150 mM NaCl, 2 mM MgCl_2_). Appropriate fractions were concentrated to 13 mg ml^–1^ (*ε* = 45 380 M^–1^ cm^–1^ was used to determine protein concentration). PPAT: 4 ml NiNTA beads were added to the cleared lysate. Elution fraction was dialysed against 50 mM Tris·HCl, pH 8.0, 150 mM NaCl and further purified by SEC (50 mM Tris·HCl, pH 7.4, 150 mM NaCl, 2 mM MgCl_2_). Appropriate fractions were concentrated to 20 mg ml^–1^ (*ε* = 8480 M^–1^ cm^–1^ was used to determine protein concentration). GB1-PCP7_tei_ and GB1-PCP7_cep_ were purified according to Haslinger and Peschke *et al.* ^36^

#### Synthesis of tyrosine-coenzyme A

Boc-l-Tyr (36 μmol, 1.5 eq.), HCTU (*O*-(6-chlorobenzotriazol-1-yl)-*N*,*N*,*N*′,*N*′-tetramethyl-uronium hexafluorophosphate, 33.6 μmol, 1.4 eq.) and HOBt (1-hydroxybenzotriazole, 33.6 μmol, 1.4 eq.) were dissolved in dimethylformamide (DMF) and diisopropylethylamine (DIPEA) (96 μmol, 4 eq.) was added. The reaction was allowed to proceed at room temperature (RT) for 10 min before coenzyme A (24 μmol, 1 eq.) was added. The reaction was then stirred overnight at RT. Boc deprotection was achieved by addition of 4 ml of trifluoroacetic acid (TFA)/H_2_O/triisopropylsilane (TIPS) (95%/2.5%/2.5%) at 4 °C, after which the solution was stirred at RT for 1.5 hours. The product was precipitated by the addition of cold diethyl ether and purified using preparative HPLC on a Waters Xbridge BEH300 Prep C_18_ column (5 μm, 19 × 150 mm) at 20 ml min^–1^ (LCMS-2020, Shimadzu, Gradient: 5% MeCN (acetonitrile) for 5 min, 5–60% MeCN in 18 min). Analysis of the purification was performed using an XBridge BEH300 C_18_ column (5 μm, 4.6 × 250 mm) at 1 ml min^–1^ (Gradient: 5% MeCN for 4 minutes, 5–10% MeCN in 11 minutes). HRMS (ESI): *m*/*z* calculated for C_30_H_45_N_8_O_18_P_3_S [M + 2H]^2+^ 464.0818, found 464.0817 (*Δ* = 0.2 ppm).

#### Synthesis of pantetheine-dipeptides

Fmoc-d-tyrosine-*O*-(2-chlorotrityl) alpha allyl ester resin (0.3 mmol, 1 eq.) was swollen in 3 ml DMF for 15 minutes, followed by Fmoc deprotection using 4 ml of 2% 1,8-diazabicyclo[5.4.0]undec-7-ene (DBU) in DMF for 15 minutes with gently shaking. The resin was washed 5 times with 6 ml DMF and 5 times with 6 ml dichloromethane (DCM). Fmoc-d-Hpg-OH (0.6 mmol, 2 eq.), HCTU (1.8 mmol, 6 eq.), HOBt (1.8 mmol, 6 eq.) and DIPEA (9 eq.) in DMF were added and the reaction mixture was left overnight at RT with gently shaking. The resin was then washed 5 times with 6 ml DMF and 5 times with 6 ml DCM. Fmoc deprotection was achieved by adding 4 ml 2% DBU in DMF to the resin for 15 minutes with gently shaking. Allyl deprotection was performed by adding Pd(Ph_3_P)_4_ (0.03 mmol, 0.1 eq.) and PhSiH_3_ (12 mmol, 40 eq.) dissolved in 3.5 ml anhydrous THF to the reaction mixture for 20 minutes with gently shaking. The resin was washed 5 times with 6 ml DMF and 5 times with 6 ml DCM. Subsequently, PyBOP (benzotriazol-1-yloxy)tripyrrolidinophosphonium hexafluoro-phosphate (0.4 mmol, 3 eq.), HOBt (0.9 mmol, 3 eq.) dissolved in 1 ml DMF and DIPEA (9 eq.) were added to the resin for 10 minutes for activation, followed by the addition of d-pantetheine (0.05 mmol, 0.2 eq.) dissolved in 1 ml DMF with gently shaking for 1.5 hours at RT. The resin was washed 5 times with 6 ml DMF and 5 times with 6 ml DCM. The peptide was cleaved with 3 ml of cleavage mixture containing acetic acid/trifluoroethanol/DCM (1 : 1 : 3) for 1.5 hours with gentle shaking, the peptide precipitated by addition of cold diethyl ether, pelleted using centrifugation before being taken up in a solution of 1 : 1 MeCN/water and lyophilised to afford a white powder. Analysis was performed using an XBridge BEH300 C_18_ column (5 μm, 4.6 × 250 mm) at 1 ml min^–1^ (gradient: 5% MeCN for 4 minutes, 5–75% MeCN in 21 minutes, *t*_R_ = 12.4 minutes). HRMS (ESI): *m*/*z* calculated for C_28_H_37_N_4_O_8_S [M – H]^–^ 589.2338, found 589.2332 (Δ*m* = 1 ppm).

#### Synthesis of chlorinated dipeptide methylamide standards

Solid phase peptide synthesis was performed manually on 0.05 mmol scale using 4-Fmoc-hydrazinobenzoyl AM NovaGel resin (80 mg, 0.62 mmol g^–1^). Fmoc deprotection was performed using a solution of 1% DBU in DMF (2 ml, 2 × 2 min). Fmoc amino acids were coupled using (1-cyano-2-ethoxy-2-oxoethylidenaminooxy)dimethylamino-morpholino-carbenium hexafluorophosphate (COMU, 4 eq.) and TEA (4 eq.); reaction times used were 12 hours for Fmocl/d-ClTyr-OH and 30 minutes for Boc-d-Hpg-OH. The displacement of the Boc-dipeptides with methylamine was achieved by adding aqueous solutions of 40% methylamine (10 eq., 43 μl) and CuSO_4_ (0.5 eq., 250 μl water) to the resin (pre-swelled in 5 ml of DMF); the reaction was allowed to proceed for 12 hours and constantly sparged with air. Afterwards, the sample was filtered through celite and the resin thoroughly washed with DMF. After removal of the DMF under reduced pressure, deprotection was achieved by addition of 5 ml of TFA/H_2_O/TIPS (95%/2.5%/2.5%) for 1 hour. The solution was then concentrated under a stream of nitrogen to ∼1 ml, the peptide precipitated by addition of cold diethyl ether, pelleted using centrifugation before being taken up in a solution of 1 : 1 MeCN/water and lyophilised to afford a white powder. This was purified by preparative HPLC on a Waters Xbridge BEH300 Prep C_18_ column (5 μm, 19 × 150 mm) at 20 ml min^–1^ (LCMS-2020, Shimadzu, gradient: 5% MeCN (acetonitrile) for 5 minutes, 5–35% MeCN in 30 minutes). H-(d)-Hpg-(d)-ClTyr–NH–Me (0.8 mg; yield% = 4.2%); MS (ESI): *m*/*z* calculated for C_28_H_37_N_4_O_8_S [M + H]^+^ 378.11, found 378.10. H-(d)-Hpg-(l)-ClTyr–NH–Me (1.2 mg; yield% = 6.3%); MS (ESI): *m*/*z* calculated for C_28_H_37_N_4_O_8_S [M + H]^+^ 378.11, found 378.10. HPLC analysis was performed using an XBridge BEH300 C_18_ column (5 μm, 4.6 × 250 mm) at 1 ml min^–1^ (gradient: 5% MeCN for 4 minutes, 5–65% MeCN in 30 minutes, (d,d)-dipeptide *t*_R_ = 9.85 minutes, (d,l)-dipeptide *t*_R_ = 10.20 minutes).

#### Synthesis of chlorinated dipeptide Leu–OH–Cl-Tyr standards

Boc-(d)-leucine (24.5 mg, 0.105 μmol) and HOBt (14 mg, 0.105 μmol) were dissolved in 500 μl of DMF at room temperature. After cooling to 0 °C, the addition of DIC (16.2 μl, 0.105 μmol) enabled the pre-activation of Boc-(d)-leucine as the-OBt ester after 30 min, the solution was added dropwise to a solution containing either (2*R*,3*R*)-β-hydroxy-3-chlorotyrosine or (2*S*,3*R*)-β-hydroxy-3-chlorotyrosine (25 mg, 0.105 μmol) dissolved in 500 μl of DMF. The pH was adjusted by the addition of TEA (36 μl, 0.26 μmol) to allow the dipeptide formation to proceed at room temperature. After 15 h, the reaction was quenched by the addition of an aqueous solution of saturated NaHCO_3_ and then acidified to pH 3 with an aqueous solution of 0.1 N HCl. The dipeptide was extracted with ethyl acetate (3×), the organic phase washed with brine (3×), dried over Na_2_SO_4_, filtered and concentrated under vacuum. The Boc protecting group was cleaved in 2 ml of a TFA/TIS/H_2_O solution (95%/2.5%/2.5%) without further purification for 1 h at room temperature. Subsequently, the solution was concentrated under a stream of nitrogen to ∼0.5 ml and the peptide precipitated *via* addition of 5 ml cold diethyl ether. After centrifugation washing with diethyl ether (3×), the peptide was dissolved in 95% water/5% MeCN, analyzed by LC-MS (gradient of 5% to 35% in MeCN in 30 minutes) and purified by RP-HPLC (gradient of 0% to 30% in MeCN in 30 minutes). H-(*R*)-Leu-(2*R*,3*R*)-β-hydroxy-3-chlorotyrosine: expected molecular mass 344.11 Da (chemical formula: C_15_H_21_ClN_2_O_5_); LC-MS analysis: rt 8.63 min ([M + H]^+^ = 345.05 Da); HRMS analysis [M + H]^+^ expected 345.1217, found 345.1208, *Δ* = 2.6 ppm. H-(*R*)-Leu-(2*S*,3*R*)-β-hydroxy-3-chlorotyrosine expected molecular mass 344.11 Da (chemical formula: C_15_H_21_ClN_2_O_5_); LC-MS analysis: rt 10.50 min ([M + H]^+^ = 345.05 Da); HRMS analysis [M + H]^+^ expected 345.1217, found 345.1209, *Δ* = 2.3 ppm.

#### Substrate loading on PCP domains

Coenzyme A coupled tyrosine was loaded on PCP domains using 1 : 3 : 0.004 ratio of PCP domain, CoA-Tyr and Sfp R4-4 (ref. 65) respectively (10 min, RT, 25 mM Tris, pH 7.4, 5 mM MgCl_2_, 1 mM DTE). For hexa- and heptapeptide-CoAs^41,42^ ratio of 1 : 3 : 10 was used (1 hour, 30 °C). Dipeptide substrates were loaded to Trx-PCP2-domain in a modified one pot loading reaction.^65,66^ 100 μM Trx-PCP2, 2.5 μM PanK and 300 μM pantetheine-dipeptide were mixed in loading buffer (100 mM Tris·HCl, pH 7.4, 5 mM MgCl_2_, 1 mM DTE). Reaction was started by addition on 1 mM ATP (5 minutes, RT). 10 μM PPAT were added and reaction incubated for another 10 minutes followed by addition of 0.1 U ml^–1^ of alkaline phosphatase (10 minutes, M0290, Roche). Finally, the substrate was loaded to Trx-PCP2 domain using 3 μM Sfp R4-4 (15 minutes).

Excess of substrate was removed after all loading reactions in centrifugal concentrators using serial concentrating-diluting steps (10 000 MWCO, 0.5 ml, Merck Millipore). Loaded PCP domains were used as quickly as possible for assays to prevent substrate hydrolysis.

#### Halogenase activity assay

9 μM Halogenase and 5.8 μM YcnD were mixed in assay buffer (50 mM Tris, pH 7.4, 10 mM NaCl, 1 mM DTE) containing FAD and FMN in excess. FAD is required by the halogenase enzyme and FMN by the reduction partner. 50 μM of substrate (either in solution or loaded to a PCP domain) was added and reactions started with 2 mM NADH (30 °C, 1 hour). 2 mM NADH was added again 15 minutes after beginning of the reaction. Substrates were cleaved from PCP-domains by addition of methylamine (3 μl, 10 minutes) and proteins precipitated with formic acid (1.5 μl). Supernatants were filtered and analysed on a Acquity UPLC Peptide BEH C18 column (1.7 μM, 2.1 × 100 mm, Waters) using Ultra High Performance Liquid Chromatography/Mass Spectrometer (LCMS-8050, Shimadzu) with single ion monitoring (SIM) and multiple reaction monitoring (MRM) (Table 2). (Gradient for analysing amino acids: 5% MeCN for 0.5 minutes, 5–10% MeCN in 3.5 minutes, for dipeptide: 5% MeCN for 0.8 minutes, 5–40% MeCN in 6.2 minutes, and for hexa/heptapeptide: 5% MeCN for 0.5 minutes, 0.5–20% MeCN in 0.2 minutes, 20–70% MeCN in 5.3 minutes).

**Table 2:** *m*/*z* values used for UPLC/MS analysis of turnover products. SIM = single ion monitoring, MRM = multiple reaction monitoring

Compound	SIM	MRM
Tyr		182 ⇒ 91
Me–NH–Tyr		195 ⇒ 91
Cl-Tyr		216 ⇒ 134
MeNH–Cl–Tyr		229 ⇒ 170
Tyr–Hpg	331 (+)	
MeNH–Tyr–Hpg	344 (+)	
Cl–Tyr–Hpg	365	
MeNH–Cl–Tyr–Hpg	378	
Type I hexapeptide (V6P)	868 (–)	
MeNH–V6P	881 (–)	
Cl–V6P	902 (–) 904 (+)	
MeNH–Cl–V6P	917 (+)	
Di-Cl-V6P	938 (+), 936 (–)	
MeNH-di-Cl-V6P	950 (+), 949 (–)	
Type I heptapeptide (V7P)	1017 (–)	
MeNH-T7P	1030 (–)	
Cl–V7P	1053 (+), 1051 (–)	
MeNH–Cl–V7P	1066 (+), 1064 (–)	
Di-Cl-V7P	1087 (+), 1085 (–)	
MeNH-di-Cl-V7P	1100 (+), 1098 (–)	
Type IV heptapeptide (T7P)	1088.5 (–)	
MeNH-T7P	1101.4 (–)	
Cl-T7P	1124.4 (+), 1122.5 (–)	
MeNH–Cl-T7P	1137.4 (+), 1135.5 (–)	
Di-Cl-T7P	1158.4 (+), 1156.4 (–)	
MeNH-di-Cl-T7P	1171 (+), 1169 (–)	
