# Supplementary material for: Halogenation of glycopeptide antibiotics occurs at the amino acid level during non-ribosomal peptide synthesis
Source: Chem Sci. 2017 Jul 13;8(9):5992–6004. doi: 10.1039/c7sc00460e (PMC5620994; doi:10.1039/c7sc00460e)
Supplement: Supplementary file 1 [file SC-008-C7SC00460E-s001.pdf]

Electronic Supplementary Material (ESI) for Chemical Science.
This journal is © The Royal Society of Chemistry 2017

Halogenation of glycopeptide antibiotics occurs at the amino acid level during non-ribosomal peptide synthesis

Tiia Kittilä,†[a] Claudia Kittel,†[b] Julien Tailhades,[c,d] Diane Butz,[f] Melanie Schoppet,[a,c,d] Anita Büttner,[e] Rob J. A. Goode,[d,i] Ralf B. Schittenhelm,[d,i] Karl-Heinz van Pee,[e] Roderich D. Süssmuth,[f] Wolfgang Wohlleben,[b,g] Max J. Cryle # [a,c,d,h] and Evi Stegmann # [b,g]

[a.] *Department of Biomolecular Mechanisms, Max Planck Institute for Medical Research, Jahnstrasse 29, 69120 Heidelberg, Germany.*
[b.] *Interfaculty Institute of Microbiology and Infection Medicine Tuebingen, Microbiology/Biotechnology, University of Tuebingen, Auf der Morgenstelle 28, 72076 Tuebingen, Germany*
[c.] *EMBL Australia, Monash University, Clayton, Victoria 3800, Australia.*
[d.] *The Monash Biomedicine Discovery Institute, Department of Biochemistry and Molecular Biology, Monash University, Clayton, Victoria 3800, Australia.*
[e.] *Allgemeine Biochemie, TU Dresden, 01062 Dresden, Germany*
[f.] *Institut für Chemie, Technische Universität Berlin, 10623 Berlin Germany*
[g.] *German Centre for Infection Research (DZIF), Partner Site Tuebingen, Tuebingen, Germany*
[h.] *ARC Centre of Excellence in Advanced Molecular Imaging, Monash University, Clayton, Victoria 3800, Australia*
[i.] *Monash Biomedical Proteomics Facility, Monash University, Clayton, Victoria 3800, Australia*

† These authors contributed equally.

# Address correspondence to: PD Dr. Evi Stegmann (evi.stegmann@biotech.uni-tuebingen.de) and A/Prof Dr. Max Cryle (max.cryle@monash.edu)

Supplementary Information

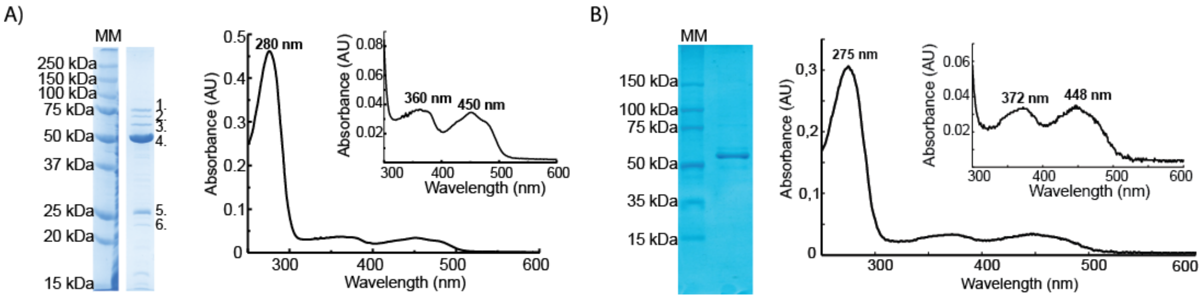

Figure S1. Purification and spectral characterisation of halogenases. A) SDS-PAGE and spectra of Tcp21, which was purified in a single affinity chromatography step with a bright yellow colour indicating the presence of FAD. Co-purifying enzymes were analysed but no reduction partner could be identified. Proteins were identified by peptide map fingerprinting as (1) Bi-functional UDP-glucuronic acid decarboxylase; (2) Glucosamine-fructose-6-phosphate aminotransferase; (3) GroEL; (4) Tcp21; (5) Peptidylprolyl isomerase; and (6) Crp/Fnr family transcriptional regulator. B) Representative SDS-PAGE and spectra of BhaA.

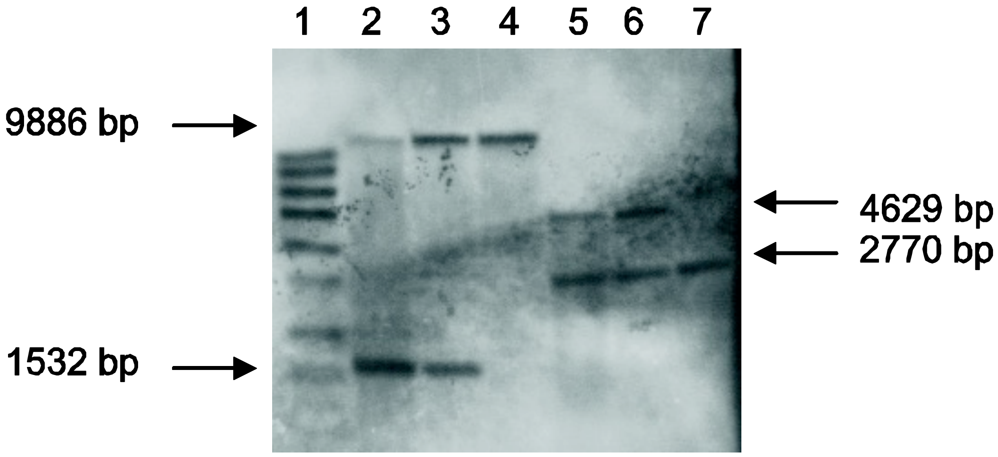

Figure S2. Southern blot analyses of wildtype and the truncation mutants CK2.1 and CK2.2. The total DNA was digested either with NcoI (N) or with BamHI. As a probe the TE domain was used, resulting in one band for the wildtype (NcoI: 9886 bp; BamHI: 2770 bp) and in two bands for the mutants (NcoI: 9886bp, 1532 bp; BamHI: 2770 bp, 4629 bp). Lane 1: DIG-labelled DNA Molecular Weight Marker VII; lane 2 and 3: mutants (NcoI), lane 4: WT (NcoI); lane 5 and 6: mutants (BamHI) and lane 7: WT (BamHI).

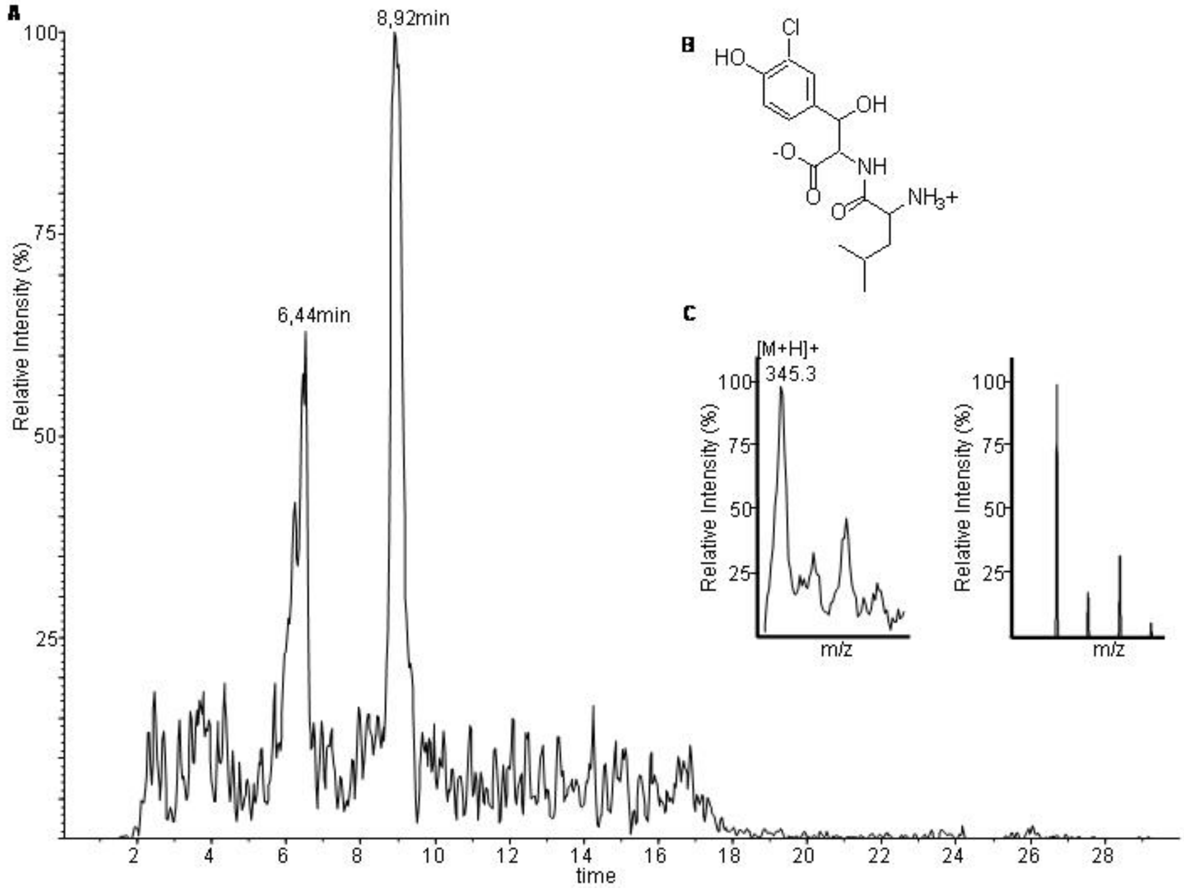

Figure S3. (A) Extracted ion chromatogram (EIC, smoothed) shows two retention times of *m/z* 345.3 of the chlorinated dipeptides. (B) Structure formula of the chlorinated dipeptide. (C) The isotopic pattern found in the spectrum (left) in comparison to the theoretically calculated isotopic pattern (right).

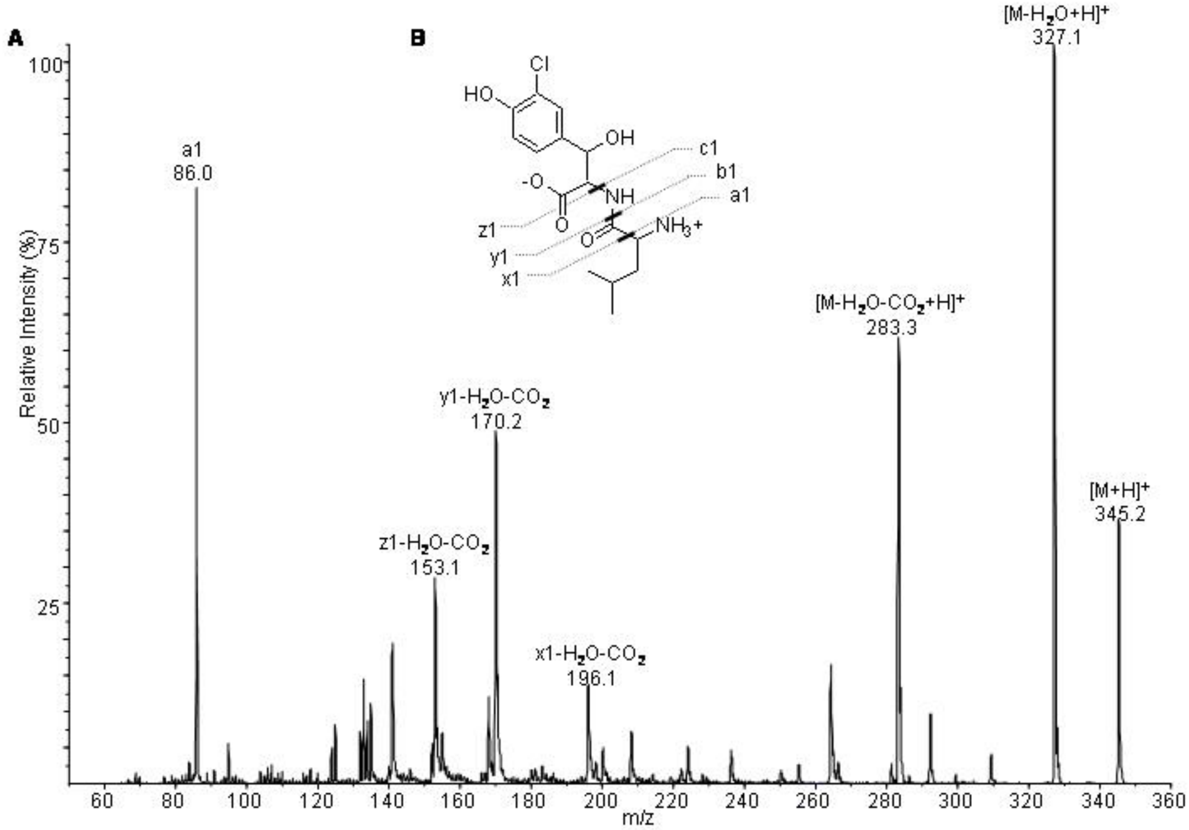

Figure S4. (A) Daughter ion spectrum of the chlorinated dipeptide (retention time: 8.92 min) and (B) assignment of the Roeppstorff fragmentation along the peptide chain.

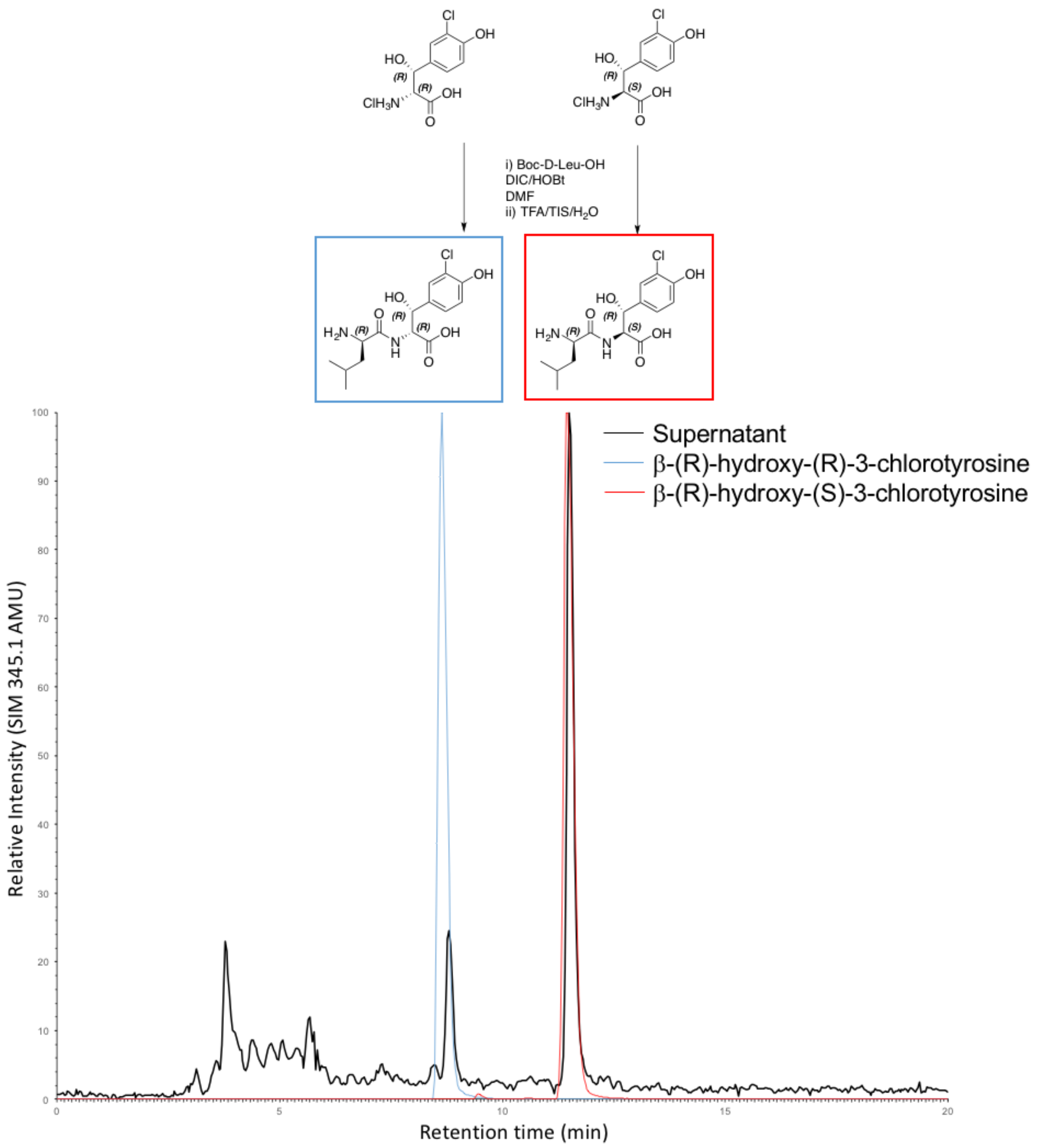

Figure S5. LC traces of the culture filtrate of the *A. balhimycina* mutant strain (black) overlayed with the traces of the authentic Leu-OH-Cl-Tyr dipeptide standards containing either (2R, 3R)-β-hydroxy-3-chlorotyrosine (blue) or (2S, 3R)-β-hydroxy-3-chlorotyrosine (red). Traces shown are in single ion monitoring mode (SIM) at 345.1 AMU, positive mode. Co-injection of synthetic Leu-OH-Cl-Tyr dipeptide standards and supernatant confirmed the retention time of the standards and dipeptide produced by the mutant strain are identical.

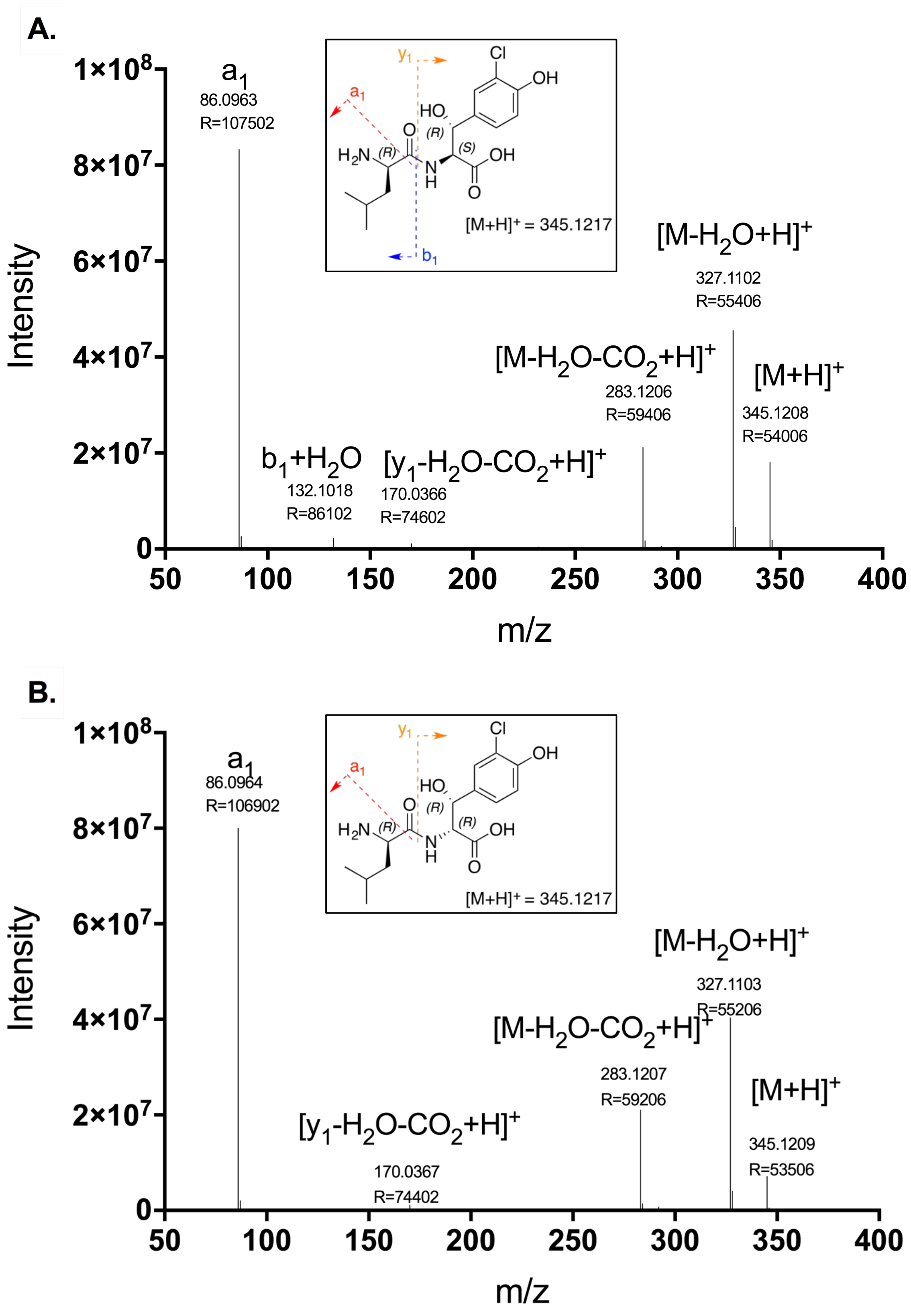

Figure S6. MS^2 fragmentation of authentic Leu-OH-Cl-Tyr dipeptide standards containing (2S, 3R)-β-hydroxy-3-chlorotyrosine (A) or (2R, 3R)-β-hydroxy-3-chlorotyrosine (B) with fragmentation pattern displayed for each dipeptide (boxed).

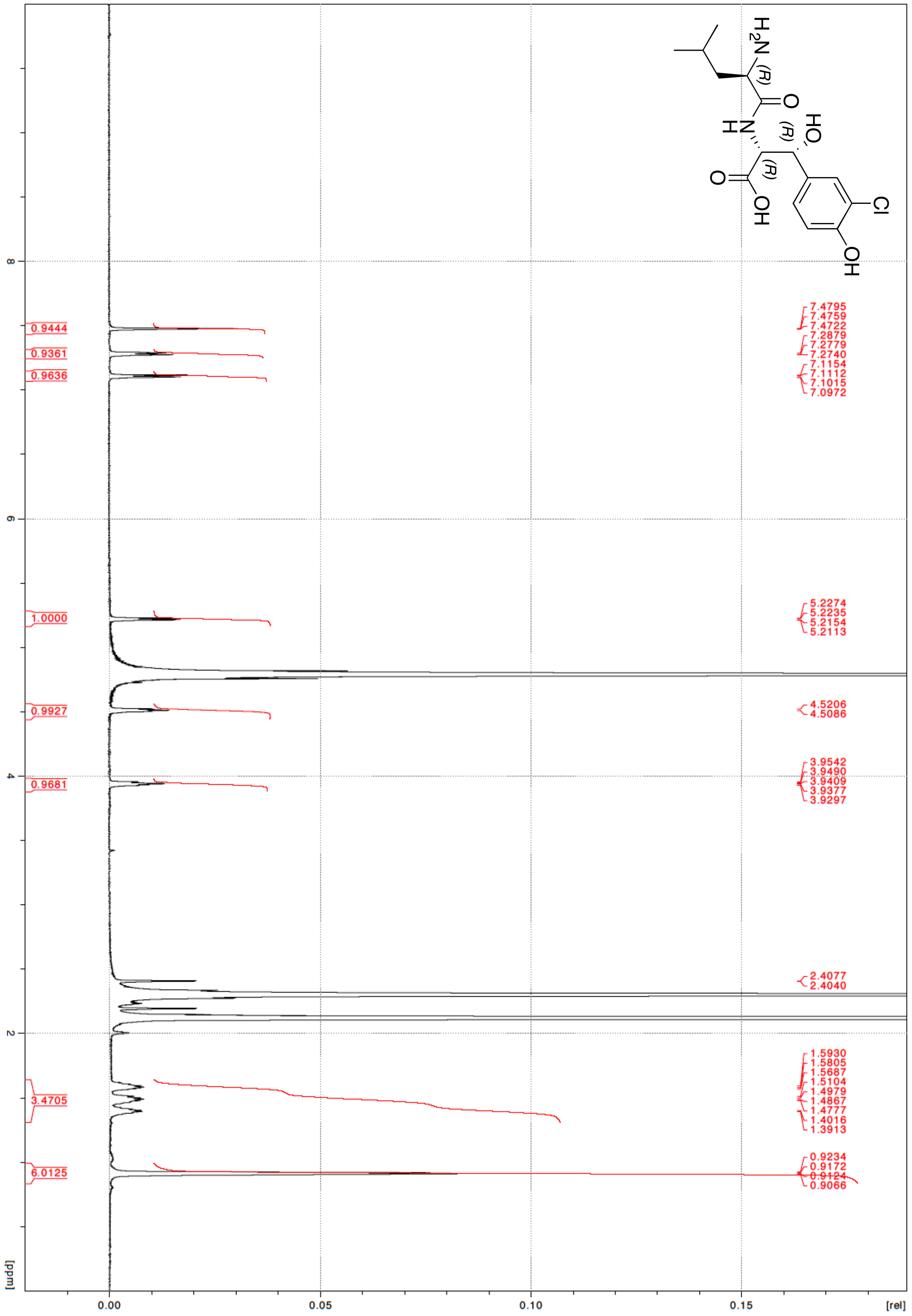

Figure S7. ^{1}H NMR (600MHz; D_2O) of authentic Leu-OH-Cl-Tyr dipeptide standard containing (2R, 3R)-β-hydroxy-3-chlorotyrosine.

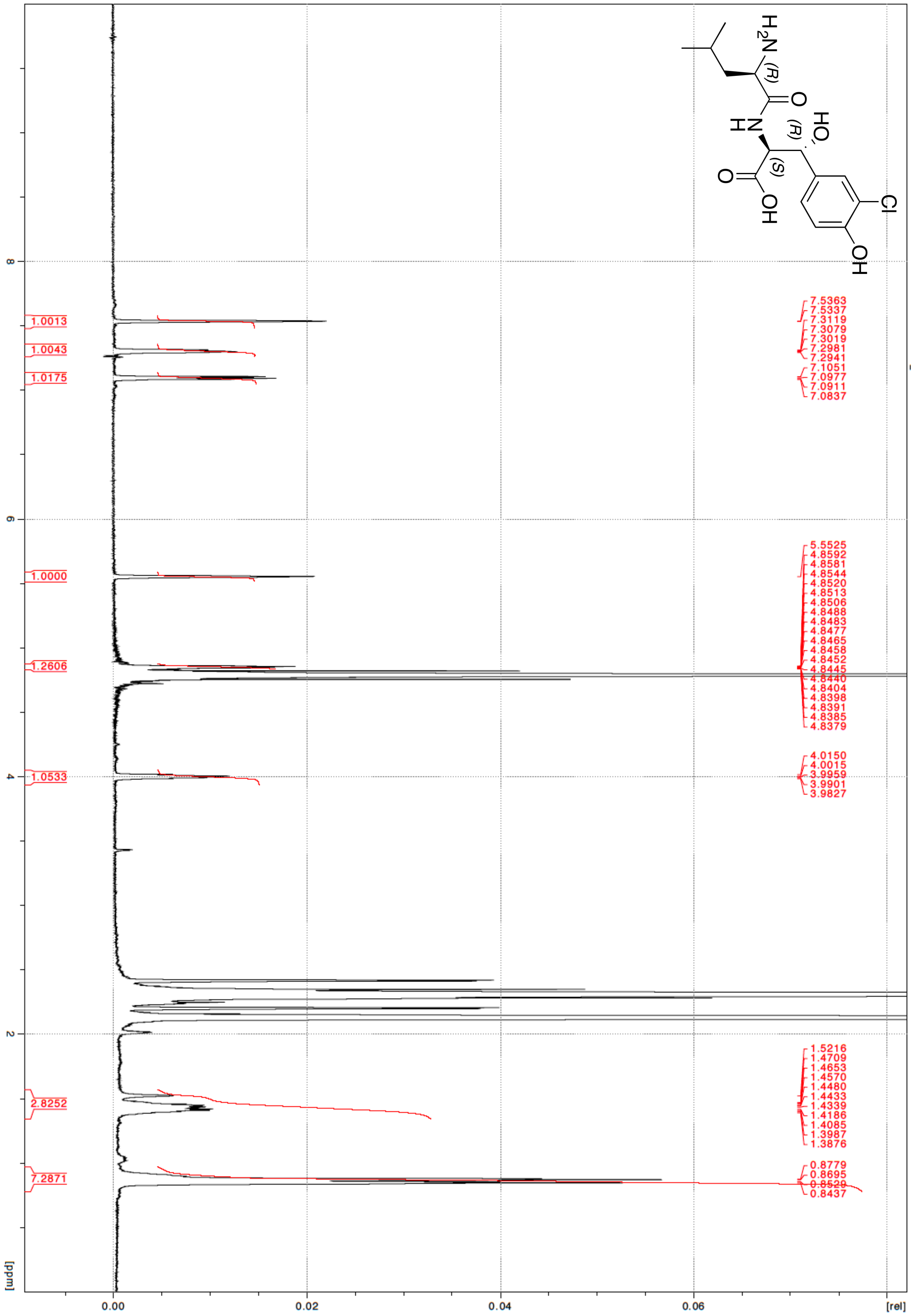

Figure S8. ^{1}H NMR (600MHz; D_2O) of authentic Leu-OH-Cl-Tyr dipeptide standard containing (2S, 3R)-β-hydroxy-3-chlorotyrosine.
